# Invasive and Subcutaneous Infections Caused by Filamentous Fungi: Report from a Portuguese Multicentric Surveillance Program

**DOI:** 10.3390/microorganisms10051010

**Published:** 2022-05-11

**Authors:** Cristina Veríssimo, Cristina Toscano, Teresa Ferreira, Gabriela Abreu, Helena Simões, José Diogo, Dinah Carvalho, Felicidade Santiago, Ana Lima, Ana Maria Queirós, Raquel Sabino

**Affiliations:** 1 Reference Unit for Parasitic and Fungal Infections, Department of Infectious Diseases, National Institute of Health, Dr. Ricardo Jorge, 1649-016 Lisbon, Portugal; helena.simoes@insa.min-saude.pt (H.S.); raquel.sabino@insa.min-saude.pt (R.S.); 2 Microbiology Laboratory, Hospital Egas Moniz, Centro Hospitalar Lisboa Ocidental, 1349-019 Lisbon, Portugal; ctoscano@chlo.min-saude.pt; 3 Centro Hospitalar Lisboa Central, 1349-019 Lisbon, Portugal; teresa.ferreira@gmail.com; 4 Centro Hospitalar Vila Nova de Gaia/Espinho, EPE, 4434-502 Vila Nova de Gaia, Portugal; mgabiabreu@gmail.com; 5 Microbiology Laboratory, Hospital Garcia de Orta, 2805-267 Almada, Portugal; jmcdiogo1@gmail.com; 6 Microbiology Laboratory, Centro Hospitalar Lisboa Norte, 1649-035 Lisbon, Portugal; dinahjcarvalho@gmail.com; 7 Centro Hospitalar de Leiria, 2410-197 Leiria, Portugal; felicidadesantiago@hotmail.com; 8 Microbiology Laboratory, Centro Hospitalar de entre Douro e Vouga, 4520-211 Santa Maria da Feira, Portugal; ana.lima@chedv.min-saude.pt; 9 Microbiology Laboratory, Centro Hospitalar Tondela Viseu, 3460-525 Viseu, Portugal; anabentoqueiros@hotmail.com; 10 Instituto de Saúde Ambiental, Faculdade de Medicina, Universidade de Lisboa, 1649-028 Lisbon, Portugal

**Keywords:** invasive fungal infections, subcutaneous fungal infections, fungal epidemiology, surveillance

## Abstract

Invasive fungal infections (IFI) have significantly increased over the past years due to advances in medical care for the at-risk immunocompromised population. IFI are often difficult to diagnose and manage, and can be associated with substantial morbidity and mortality. This study aims to contribute to understanding the etiology of invasive and subcutaneous fungal infections, their associated risk factors, and to perceive the outcome of patients who developed invasive disease, raising awareness of these infections at a local level but also in a global context. A laboratory surveillance approach was conducted over a seven-year period and included: (i) cases of invasive and subcutaneous fungal infections caused by filamentous/dimorphic fungi, confirmed by either microscopy or positive culture from sterile samples, (ii) cases diagnosed as probable IFI according to the criteria established by EORTC/MSG when duly substantiated. Fourteen Portuguese laboratories were enrolled. Cases included in this study were classified according to the new consensus definitions of invasive fungal diseases (IFD) published in 2020 as follows: proven IFI (N = 31), subcutaneous fungal infection (N = 23). Those proven deep fungal infections (N = 54) totalized 71.1% of the total cases, whereas 28.9% were classified as probable IFI (N = 22). It was possible to identify the etiological fungal agent in 73 cases (96%). *Aspergillus* was the most frequent genera detected, but endemic dimorphic fungi represented 14.47% (N = 11) of the total cases. Despite the small number of cases, a high diversity of species were involved in deep fungal infections. This fact has implications for clinical and laboratory diagnosis, and on the therapeutic management of these infections, since different species, even within the same genus, can present diverse patterns of susceptibility to antifungals.

## 1. Introduction

Invasive fungal infections (IFI) have significantly increased over the past years due to advances in medical care to the at-risk immunocompromised population [1,2]. The number and heterogeneity of patients at risk have increased, especially due to the wider use of intensive myelosuppressive and/or immunosuppressive agents in the treatment of haematological cancers (in particular in those with acute myeloid leukaemia and myelodysplastic syndromes), the growing number of patients undergoing allogeneic haematopoietic stem cell transplantation (HSCT) and the increasing aged population [3,4]. In Europe, the number of stem cell transplantations almost doubled between 2000 and 2016 and at the same time, new at-risk populations were identified, as patients with severe influenza or chronic obstructive pulmonary disease [5].

The IFI incidence is approximately 6 cases per 100,000 persons per year [5]. Rates of IFI-related mortality in Europe depend on the pathogen, geographical location and underlying characteristics of the patients, with rates ranging from 38 to 80% for invasive aspergillosis [6]. 

The most frequent filamentous fungi (moulds) isolated from IFI are *Aspergillus* spp. [3,7,8], but *Fusarium* spp., *Scedosporium* spp. and fungi belonging to Mucorales order are increasingly seen [8,9]. Invasive fungal infections caused by these fungi are a major cause of morbidity and mortality in patients with haematological malignancies [10]. 

IFI are often difficult to detect and treat [11,12], and can be associated with substantial morbidity and mortality [13,14]. Early diagnosis can improve treatment outcomes and potentially reduce IFI-associated hospital costs [15,16,17]. National surveillance programs on IFI raise awareness of these infections, enabling the improvement of their diagnosis and treatment. Until a few decades ago, the prevalence of fungal infections was low or unknown in Portugal, which may be explained by the lack of regular national surveillance and also by the lack of an obligatory reporting system for the occurrence of these infections. In 2017, the burden of serious fungal disease in Portugal was estimated using deterministic scenario modelling, and the incidence and prevalence were calculated [18]. However, a multicentric Portuguese survey involving cases of proven and probable mould diseases was still lacking and no national study on this issue has been published so far, representing a major need at epidemiological level. Therefore, the present study, based on a laboratory surveillance approach, has been conducted over a seven-year period. This study aims to enhance the knowledge about the etiology of invasive and subcutaneous fungal infections among the participating hospital units, to understand the main risk factors associated with those infections and to perceive the outcome of patients who developed invasive disease. 

## 2. Materials and Methods

### 2.1. Cases’ Registry 

Microbiology laboratories from hospitals belonging to the national health system were invited to participate in a multicentric surveillance program on fungal infections. Each hospital unit designated a focal-point element responsible for collecting the fungal isolate (whenever possible) and for gathering clinical, laboratory and epidemiological information. These data were registered, filling out a survey that included questions related to the patients‘ demographic features, clinical and mycological criteria and also host factors for case inclusion. The survey also included questions linked to patients’ risk factors for invasive fungal infection and their outcome after 30 days of therapy. Risk factors included in the questionnaire were divided into three groups: (a) immunosuppression factors (chemotherapy, hematopoietic bone marrow transplantation, allogeneic bone marrow transplantation, solid organ transplantation, HIV/AIDS infection, another disorder requiring immunosuppression; (b) trauma/intervention (hospitalization in the ICU, invasive surgery, burn, penetrating trauma) and (c) chronic diseases/behavioral factors (alcoholism, COPD (chronic obstructive pulmonary disease), diabetes mellitus, chronic kidney/liver disease, travel to endemic fungal infections, premature birth). All the included cases were anonymized before being added to the network.

This laboratory network enrolled fourteen microbiology laboratories from hospitals located predominantly in the central and northern regions of Portugal. Nine of these fourteen centers participated actively in the present study.

### 2.2. Criteria Used for the Inclusion of Cases in This Study

From all the reported cases (from January 2013 to May 2020), the ones included for further analysis were: (i) cases of proven fungal invasive and subcutaneous infections caused by filamentous/dimorphic fungi, confirmed by microscopy, positive culture obtained from sterile samples (collected by biopsy or puncture) or by panfungal PCR, performed in tissue samples with visible fungal structures and when clinical was compatible with fungal infection; (ii) cases diagnosed as probable IFI according to the criteria established by EORTC/MSG 2020 [19] with mycological evidence of infection (filamentous fungi recovered by culture or microscopic detection of fungal elements in sputum, BAL, bronchial brush, or aspirate, detection of the antigen galactomannan, and *Aspergillus* PCR) when duly substantiated by the presence of a host factor and clinical feature. Cases of fungal infections caused by *Candida* spp., *Cryptococcus* spp. and *Pneumocystis jirovecii* were not included. 

### 2.3. Identification of the Isolated Fungi 

When a positive culture was obtained, the isolated fungi was sent to the Mycology National Reference Laboratory at the National Health Reference Dr. Ricardo Jorge (INSA), IP to confirm the identification to genus/species level. In a first step, isolated fungi were identified based on the observation of their macro and microscopic morphology according to what is described in the identification atlas [20]. To achieve and/or confirm the identification at the species level, extraction of total DNA from fungal colonies was performed, and species level identification was achieved by sequencing the ITS (internal transcribed spacer) region of the ribosomal DNA [21] or, in the case of the *Aspergillus* spp., by partial sequencing of the coding region for calmodulin [22]. The obtained sequences were then compared with sequences deposited in NCBI Blast and Westerdijk Fungal Biodiversity Institute databases. In samples with positive histology for fungal structures and from which no culture was obtained, an in house panfungal PCR or a PCR targeted to *Aspergillus* or Mucorales (Pathonostics^®^ (Maastricht, The Netherlands)) were performed, whenever possible, to identify the fungal agent [23,24].

## 3. Results

During the study period (January 2013 to May 2020), 103 cases were submitted to our surveillance program and 76 were validated, the majority of them from Lisbon and the Tagus Valley region. 

Included cases were categorized as invasive fungal infections (IFI) (N = 53) and as subcutaneous fungal infections (SFI) (N = 23) (Table 1). Overall, these infections were more frequently reported in males (N = 51) and less frequently in females (N = 25). The median age of patients was 59.5 years (ranging 3–90 years). Table 1 shows these data discriminated by type of infection (IFI and SFI). 

The validated 76 reports were distributed as follows: 54 cases of proven fungal infections from which 31 were classified as IFI and 23 as SFI, totaling 71.1% (N = 54) of proven fungal infections and 28.9% (N = 22) of probable IFI (Table 2). The obtained results show that 11 IFI cases were caused by endemic fungi. Invasive proven and probable fungal infections represented 69.7% (N = 53) of total cases. Analysis of our data revealed a predominance of localized infections (N = 66). Disseminated infections were observed only in 10 cases. From these latter, nine were classified as proven IFI, four of them were caused by endemic dimorphic fungi and one was classified as a probable IFI. 

In general, positive microscopy was detected in about half of the cases and positive cultures were obtained in 83.0 and 95.6 of IFI and SFI cases, respectively (Table 1). In eight cases (no. 25, 29, 32, 53, 64, 66, 70, 74) no positive culture was obtained and in two cases (no. 36, and 52) cultures were not performed (Table 2). These cases were included as validated cases of IFI based on the positive microscopy of the samples, collected from a sterile site. Septate/aseptate hyphae or characteristic structures of endemic fungi (*P*. *brasiliensiis* and *H. capsulatum duboisii*) were observed in those samples. In seven of those cases (no. 29, 32, 36, 52, 66, 70, 74) it was possible to confirm the identification of the etiological agent through panfungal PCR followed by sequencing. In case no. 29, only the application of real time PCR targeted to *Aspergillus* allowed the identification of the possible etiological agent, since microscopy was not performed, and culture was negative. Case no. 36 presented aseptate hyphae in direct microscopy and Mucorales infection was confirmed by real time PCR targeted to Mucorales, but this test does not allow the identification of the etiological agent. Furthermore, sample no. 64 showed aseptate hyphae, but identification of the etiological agent was not performed because the Mucorales culture was lost. Although positive fungal structures were seen in tissue from sample no. 53, culture and panfungal PCR were both negative. Thus, in 73 out of 76 cases (96%), it was possible to identify the etiological fungal agent (Table 2). 

*Aspergillus* was the most frequent fungal genera detected (N = 15; 19.7%), being more frequently identified in the proven and probable IFI. Section *Fumigati* was the most frequent section found (N =11), representing 20.8% of the etiological agents causing proven and probable IFI (N = 53). In this section, the species *A. fumigatus sensu stricto* was the most frequently detected, but *A. felis/parafelis* was also identified in one case (case no. 63). The remaining *Aspergillus* sections identified in this study were *Flavi* (N = 2), *Nigri* (N = 1) and *Nidulantes* (N = 1). 

Infections caused by the genera *Scedosporium* spp. (N = 9), *Alternaria* spp. (N = 9) and *Fusarium* (N = 6) represented, respectively, 11.8%, 11.8% and 7.9% of the validated cases. Species from the *Scedosporium apiospermum* complex and the species *Alternaria infectoria* were the most frequently detected in subcutaneous infections.

Aseptate fungi belonging to the Mucorales order (N = 7) were the cause of 9.2% of the studied infections, and the genera identified were *Mucor*, *Rhizopus*, *Cunninghamella*, *Lichteimia* and *Saksenaea*. The latter, less frequently identified, was responsible for a subcutaneous infection as a result of a penetrating trauma (case no. 35) (Table 2). 

During the study period, it was also observed that invasive fungal infections (proven and probable) were caused by rare fungal agents, namely the dematiaceous fungi *Cladophialophora bantiana* (brain abcess), several species of Mucorales (*Cunninghamella bertholetiae*, *Mucor velutinosus*, *Rhizopus microsporus*, *Lichteimia racemosa*), and several species of hyaline fungi as *Fusarium* spp. (*F. solani*, *F. dimerum. F. proliferatum*, *F. neocosmoporielum*), *Purpureocillium lillacinus* (eye infection), *Paecillomyces formosus* and *Radulidium subulatum* (respiratory infection) (Table 2). 

Infections caused by dimorphic endemic fungi represented 14.5% (N = 11) of the total cases. Those infections were all imported cases from endemic areas and were caused by species belonging to the genera *Histoplasma* (*n* = 8) and *Paracoccidioides* (N = 3) (Table 2). 

In 81.6% (N = 62) of cases, one or more risk factors for fungal infection were reported (Table 1 and Table 2). In this study, the main risk factors associated with the development of fungal infections were: immunosuppression (N = 12); invasive surgery (N = 7); alcoholism (N = 6); diabetes mellitus (N = 5), transplantation (N = 13); and acute myeloid leukemia (N = 3). In addition to travelling to endemic regions, HIV/AIDS and alcoholism are also risk factors associated with infections caused by dimorphic endemic fungi.

In 68% of the cases (N = 52), it was not possible to obtain information about the patients’ outcome. Nevertheless, in 33.3% (N = 8) of the 24 cases with reported information on that question, the fungal infection resulted in the patient death (Table 2). 

## 4. Discussion

The results obtained with this multicentric surveillance program allowed the perception of the high diversity of species identified as etiological agents of the diagnosed IFI cases. Some of those species are described as being less susceptible to antifungals [25], which may lead to difficulties in the management of those infections. It is also important to highlight that several rare species were identified as etiological agents of the deep fungal infections, as described in similar studies [26]. In immunosuppressed patients, this fact represents a major challenge in the diagnosis and treatment of these infections. Additionally, the number and heterogeneity of patients at risk for invasive fungal infections have been increasing [5], and the epidemiology of IFI and SFI varies according to the geographical location. Thus, the high intercontinental mobility increases the possibility of detecting infections caused by rare fungi, namely by endemic fungi.

In the following lines, a more detailed analysis on some of the detected fungal agents and associated infections is discussed.

### 4.1. Invasive Fungal Infections

Taking into account the etiology of the proven and probable IFI (N = 53), the etiological agent predominantly identified in all the analyzed infections is *Aspergillus* spp. (28.3%). This result is in line with the epidemiology described by the majority of the published European studies (when excluded *Candida* spp., *Cryptococcus* spp. And *Pneumocystis jiriveci*) [27,28,29]. A previous study on the burden of fungal infections in Portugal estimated that 65 cases of invasive aspergillosis (IA) occur annually in HSCT and solid organ-transplanted patients [18]; in this work, we also detected cases from other described risk patients, such as the ones hospitalized in intensive care units, with sarcoidosis or chronic pulmonary obstructive disease (COPD), as also reported in other studies [30,31,32]. In similar studies in the USA, the frequency of IA was 8.9%, whereas dimorphic fungi was 25.2% and 1.1% for Mucorales [33]. Although lower than in the USA, our data revealed a surprisingly high frequency of infections caused by dimorphic fungi (14.5%), since Portugal is not an endemic area of these fungi. The Portuguese fungal burden study [18] described an annual incidence of mucormycosis of 10 cases. In the present study, the infections caused by aseptate fungi have shown a higher frequency (9.2%) than that in the USA studies. 

The increased fungal burden and the detected species seem to be related, with the introduction of novel immunosuppressive regimens among patients undergoing bone marrow or solid organ transplants, or treatment for malignancies.

Systemic endemic mycoses are mostly found in the Americas, Africa, and Southeast Asia, where their true burden is poorly defined [34]. During the study period, histoplasmosis and paracoccidioidomycosis were the most frequently detected endemic mycoses. All these cases were imported from endemic areas. These diseases are commonly misdiagnosed as tuberculosis, resulting in a substantial delay in the treatment, being therefore considered as neglected mycoses [34]. In fact, four cases of the reported endemic mycoses resulted in disseminated infection (histoplasmosis) and in three of them, the patient became deceased. The estimated incidence of histoplasmosis is 20% for individuals who had travelled to Latin America for the first time [35]. Paracoccidioidomycosis is the second most prevalent endemic mycosis in Latin America; it is estimated that 10 million of Latin Americans are infected and that 1–2% will present with some clinical form of the disease some weeks to several decades after exposure [34]. Since Portugal is a non-endemic region, the high number of endemic cases reported in this study may be due to the high number of citizens from Latin America (especially from Brazil and African Portuguese speaking countries) living in Portugal. Moreover, according to recent data, in 2011, the estimated number of travels made from Portugal to Americas and to Africa was 2,458,900 and 1,154,000 respectively [18], which reinforces the great exchange of persons among these continents. These numbers show that endemic mycosis should also be considered in clinical diagnosis performed in Portugal. Therefore, although paracoccidiomycosis and histoplasmosis are considered as rare diseases in Portugal, information associated with endemic areas should be kept in mind for persons (immunosuppressed or not) who were born in or have travelled to endemic regions, even if the return from the disease-endemic area occurred many years before the onset of the infection [36].

### 4.2. Subcutaneous Fungal Infections

In this study, we have considered subcutaneous fungal infection as a separate group for classifying fungal disease due to their differences in the mode of infection, clinical presentation and epidemiological pattern. These are localized infections of the skin and subcutaneous tissue following a traumatic implantation of the aetiologic agent. On rare occasions, lymphatic and hematogenous spreading of the agent can occur [37]. The causative fungi are all soil saprophytes, and the epidemiology of these infections varies geographically. 

Sporotrichosis is described as the most prevalent and widespread implantation mycosis in the world [34], but *Alternaria* was the most frequently identified genus collected from subcutaneous infections in our study. In a review on alternariosis reported up to 2007, most of the cases of subcutaneous infections caused by *Alternaria* were from Mediterranean countries [38]. The main risk factors described were penetrating trauma, solid organ transplant, diabetes mellitus and immunosuppression. These infections are usually more frequent in males, which may be explained by the fact that outdoor work is carried out more frequently by males, enhancing the risk of skin trauma [38]. Contrarily to other studies [39], our results showed no differences between males and females. 

*Scedosporium* spp. are increasingly recognized as a cause of resistant life-threatening infections in immunocompromised patients [40]. Infections caused by the *Scedosporium* genus represented 13.0% (N = 3 out of 23) of the subcutaneous infections. *Scedosporium apiospermum* (complex) was the only species of this genus that was detected in the studied subcutaneous infections. This complex of species is underlined as an emerging opportunistic filamentous fungi, with several reports described [41,42]. Treatment of *Scedosporium* infections is especially challenging due to the high levels of antifungal resistance [35,43]. Yet regarding subcutaneous infections, *Trichophyton rubrum* was identified as the etiological agent of three cases of these infections. This species is an anthropophilic dermatophyte fungus, rarely described as causing deep infections. However, several studies have described deep or invasive disease caused by *T. rubrum* in immunosuppressed patients [44,45]. As in our study, all those cases were from patients subjected to solid organ transplantation. In these cases, invasion is limited mainly to the extremities, and there is subcutaneous involvement, but without involvement of other internal organs [45]. 

*Saksenae vasiformis* and *Nannizziopsis obscura* are fungal species rarely associated with infections. In our study, these species were both isolated from skin biopsies. Organ transplantation and penetrating trauma are frequently referred as risk factors for this type of infection, as occurred in our study. Other risk factors include hematologic malignancy, diabetes, and prolonged corticosteroid use [46,47,48,49]. 

Until very recently, IFI diagnosis was only based on microscopy and culture. Histological stains for fungi and culture are still the gold standard for the diagnosis of invasive fungal infections [19]. Detection of fungal biomarkers such as the *Aspergillus* galactomannan [50], 1,3 β-D-glucan and antibody detection for some endemic fungi (in few laboratories in Europe) may also provide data that contribute to IFI diagnosis [19]. The combining antibody and antigen testing enhances the sensitivity in detection histoplasmosis [51], for example. 

In the last years, an increase in the commercially available PCR methodologies for the detection of the most frequent fungi, like *Aspergillus* and Mucorales has been observed. These molecular methodologies are not available for the detection of other emerging fungi like *Scedosporium* spp, *Fusarium* spp., and *Cladophialophora* spp. Even the PCR targeted to *Mucorales* used in this study, would fail in the detection of *Saksenae vasiformis* (isolated from a subcutaneous sample after a penetrating trauma). The commercial kit for Mucorales DNA detection used in this study allows the detection (but not the identification) of only of the following genera: *Mucor*, *Cunninghamella*, *Rhizopus*, *Rhizomucor* and *Lichteimia*. *Saksenae vasiformis* was only possible to identify by ITS sequencing of the obtained culture.

Positive cultures were obtained in the majority of the included cases, with the exception of 10. Those cases were included and validated by the observation of fungal structures in samples collected from a usually sterile site. Panfungal PCR followed by sequencing or PCRs targeted to *Aspergillus*/Mucorales were performed whenever possible, and allowed the identification of the etiological agent in two cases. This fact highlights the essential role of molecular techniques for the detection and identification of IFI agents and that polyphasic approach using several methodologies increases the efficiency of the detection and identification of IFI.

In December 2019, a new guideline on invasive fungal infections was published [19], presenting several changes to the previous guideline. In the new definition of invasive fungal disease, a positive PCR amplification followed by sequencing (applied to paraffinized tissues with histological evidence of infection by filamentous fungi) prove the invasive fungal infection by mold, when in the presence of clinical criteria compatible with infection. Microscopic observation of typical and unique structures of endemic fungi is now considered as a criterion of proven IFI as well. Hence, the revision of the criteria according to these new recommendations increased the number of proven IFI due to the reclassification of some cases, initially classified (in the beginning of this study) according to the previous guidelines.

This study presents several limitations, namely the lack of data on the antifungal susceptibility of the obtained isolates. The representativeness of this work could also be affected by several factors, especially associated with the guideline followed for the inclusion of cases, which was designed targeted to the group of hematological patients, and may therefore not be suitable for critical patients in intensive care units. It is also important to emphasize that the presented results may not reflect the Portuguese reality on deep fungal infections that are probably underrepresented. A higher number of cases were recorded in large urban areas, such as Lisbon and Oporto, where hospitals with a larger number of transplants and critically ill patients predominate. However, data on fungal infections from some regions of the country are lacking. This may contribute to the small number of analyzed cases. Difficulties on laboratory diagnosis of these infections, as the low sensitivity of conventional methods, and the low number of autopsies performed may have also contributed the missing of several IFI cases. 

## 5. Conclusions

This study presents the data on deep fungal infections from a multicentric surveillance program in Portugal, for a seven-year period. 

Despite the small number of cases, a high diversity of species involved in deep fungal infections was found during the study period. This fact has implications concerning clinical and laboratory diagnosis and the treatment of these infections, since different species/genera of fungi can present different patterns of susceptibility to antifungals, some of which are resistant to more than one class of antifungals. The surveillance of these infections is therefore essential, along with training in mycology, namely for pathologists, infectious diseases specialists and microbiology laboratory technicians.

## Figures and Tables

**Table 1:** Overall characterization of the 76 cases included in this study.

	Gender	Age	Positive Microscopy	Positive Culture	Most Common Risk Factors
	Male (N; %)	Median (Range)	(N; %)	(N; %)	
IFI (N = 53)	41; 77.3	61 (3–84)	27; 50.9	44; 83.0	Immunosuppression not associated with transplantation or HIV
Subcutaneous (N = 23)	10; 43.5	61 (7–90)	13; 56.5	22; 95.6	Solid organ transplantation and penetrating trauma

Legend: IFI: invasive fungal infection; SFI: subcutaneous fungal infection.

**Table 2:** Characterization of the validated IFI and SFI cases enrolled in the multicentric surveillance program in the period 2013–2020.

Case No.	Gender	Age	Biological Sample	Microscopy	Culture	Identification of the Etiological Agent	Classification of the Infection	Risk Factors for Fungal Infection	Outcome
1	M	40	Blood	Compatible with *Histoplasma capsulatum*	Positive	*Histoplasma capsulatum capsulatum*	Proven IFI Disseminated	HIV/AIDS; travels to areas with endemic fungi	Patient’s death due to fungal infection
2	M	64	Jugal mucosa and BAL	Compatible with *Paracoccidioides brasiliensis*	Positive	*Paracoccidoides brasiliensis*	Proven IFI Localized	Alcoholism; travels to areas with endemic fungi	Partial response to antifungal treatment
3	F	52	Tissue and BAL	Septate hyphae	Positive	*Scedosporium apiospermum* (complex)	Proven IFI Localized	Invasive surgery, imunossupression	Partial response to antifungal treatment
4	M	48	BAL	Septate hyphae	Positive	*A. fumigatus* (sensu stricto)	Probable IFI Localized	Solid organ transplant; invasive surgery; alcoholism, chronic liver disease	Patient’s death due to fungal infection
5	M	76	Skin	Yeasts	Positive	*Histoplasma capsulatum duboisii*	Proven IFI Localized	HIV/AIDS; travels to areas with endemic fungi	Partial response to antifungal treatment
6	M	65	Skin (both leg/hand)	Septate hyphae	Positive	*Alternaria infectoria*/ *Alternaria alternata*	Proven Subcutaneous Localized	Diabetes mellitus; immunosuppression	Good response to antifungal treatment
7	M	29	Bone marrow	Yeasts	Positive	*Histoplasma capsulatum capsulatum*	Proven IFI Disseminated	HIV/AIDS; travels to areas with endemic fungi	Patient’s death due to fungal infection
8	M	77	BAL	NP	Positive	*Aspergillus fumigatus* (sensu stricto)	Probable IFI Localized	Immunosuppression; intensive care hospitalization	NA
9	M	56	Brain abcess	NA	Positive	*Cladophialophora bantiana*	Proven IFI Localized	Alcoholism, drug abuse, chronic liver disease	Improvement
10	M	7	Skin	Septate hyphae	Positive	*Schizophyllum commune*	Proven Subcutaneous Localized	Trauma	Partial response to antifungal treatment
11	M	56	Pleural fluid	Aseptate hyphae	Positive	*Cunninghamella bertholetiae*	Probable IFI Localized	Trauma, alcoholism	NA
12	F	47	Skin	Fungal elements	Positive	*Sporothrix schenckii* (complex)	Proven Subcutaneous Localized	NA	NA
13	M	64	Colon	Yeasts	Positive	*Histoplasma capsulatum duboisii*	Proven IFI Localized	Alcoholism, travels to areas with endemic fungi	NA
14	M	3	Sputum/urine	NP	Positive	*Mucor velutinosus*	Probable IFI Disseminated	Intensive care hospitalization; invasive surgery; early birth	Good response to antifungal treatment
15	M	67	BAL	Negative	Positive	*Aspergillus fumigatus* (sensu stricto)	Probable IFI Localized	COPD	NA
16	M	29	Skin	Negative	Positive	*Trychophyton verrucosum*	Proven Subcutaneous Localized	Trauma (cattle breeder)	Good response to antifungal treatment
17	M	68	Tonsil	Yeasts	Positive	*Histoplasma capsulatum duboisii*	Proven IFI Localized	Alcoholism; travels to areas with endemic fungi	Good response to antifungal treatment
18	F	59	Skin	Septate hyphae	Positive	*Alternaria infectoria*	Proven Subcutaneous Localized	Solid organ transplant	NA
19	M	81	Skin	Yeasts	Positive	*Trichosporon montevidense*	Proven Subcutaneous Localized	NA	NA
20	F	75	Skin	NP	Positive	*Scedosporium apiospermum* (complex)	Proven Subcutaneous Localized	Imunossupression	Good response to antifungal treatment
21	F	61	Skin	Compatible with *Blastomyces*	Positive	*Alternaria infectoria*	Proven Subcutaneous Localized	NA	NA
22	M	90	Skin	Septate hyphae	Positive	*Scedosporium apiospermum* (complex)	Proven Subcutaneous Localized	Immunosuppression (lung cancer)	NA
23	M	29	BAL/Thraqueal lesion	Septate hyphae	Positive	*Radulidium subulatum*	Probable IFI Localized	HIV/AIDS; travels to areas with endemic fungi	NA
24	F	42	BAL	NP	Positive	*Scedosporium apiospermum* (complex)	Probable IFI Localized	Solid organ transplant, COPD	NA
25	M	72	Skin	Large yeasts	Negative	*H. capsulatum duboisii*	Proven IFI Localized	Chronic kidney disease; travel to areas with endemic fungi; immunossupression	NA
26	M	51	BAL	NA	Positive	*Scedosporium aurantiacum*	Probable IFI Localized	Organ transplant; HIV/AIDS	NA
27	M	86	Sphenoid bone	NA	Positive	*Scedosporium apiospermum* (complex)	Proven IFI Localized	NA	NA
28	F	78	BAL	NA	Positive	*A. fumigatus* (Section)	Probable IFI Localized	Invasive surgery	Patient’s death due to fungal infection
29	M	14	Nasal tissue	NP	Negative	*Aspergillus fumigatus*	Probable IFI Localized	Bone marrow transplant	Good response to antifungal treatment
30	F	75	Skin	septate hyphae	Positive	*Alternaria alternata*	Proven Subcutaneous Localized	Penetrating trauma	NA
31	F	63	Skin	NP	Positive	*Alternaria infectoria*	Proven Subcutaneous Localized	Imunossupression	NA
32	M	47	Lip tissue	Compatible with *Paracoccidioides brasiliensis*	Negative	*Paracoccidioides brasiliensis*	Proven IFI Disseminated	Alcoholism; travels to areas with endemic fungi	Good response to antifungal treatment
33	M	74	Skin	NP	Positive	*Scedosporium apiospermum* (complex)	Proven Subcutaneous Localized	Chemotherapy; invasive surgery	Relapse
34	M	68	Sphenoid bone	Aseptate hyphae	Positive	*Rhizopus microsporus*	Proven IFI Disseminated	Diabetes mellitus	Patient’s death due to fungal infection
35	F	80	Skin	NP	Positive	*Saksenae vasiformis*	Proven Subcutaneous Localized	Penetrating trauma	NA
36	M	61	Eye tissue	Aseptate hyphae	NP	Not identified Mucorales	Proven IFI Localized	Invasive surgery (eye)	NA
37	F	55	BAL	NP	Positive	*Sedosporium boydii*	Proven IFI Localized	Solid organ transplant	NA
38	F	79	Skin	NA	Positive	*Alternaria infectoria*	Proven Subcutaneous Localized	Chemotherapy	NA
39	M	54	Bone	NP	Positive	*Trichosporon mucoides*	Proven Subcutaneous Localized	Invasive surgery	NA
40	F	48	Finger	NP	Positive	*Fusarium solani* (complex)	Proven Subcutaneous Localized	NA	NA
41	F	51	Blood culture	NP	Positive	*Fusarium dimerum*	Proven IFI Disseminated	NA	NA
42	M	64	Bronchial aspirate	NP	Positive	*Exophiala* spp.	Probable IFI Localized	Imunossupression	NA
43	M	53	BAL	NP	Positive	*Aspergillus flavus*	Probable IFI Localized	HIV/AIDS	NA
44	M	69	Periorbital exsudate	NP	Positive	Not identified Mucorales	Probable IFI Localized	Diabetes mellitus	Patient’s death due to fungal infection
45	M	65	Brain abcess	Septate hyphae	Positive	*Aspergillus fumigatus* (sensu stricto)	Proven IFI Localized	Diabetes mellitus	Patient’s death due to fungal infection
46	F	40	Nasal exsudate	NP	Positive	*Fusarium proliferatum*	Probable IFI Localized	Imunossupression (acute myeloid leukemia)	NA
47	M	52	Stump tissue (traumatic amputation)	NP	Positive	*Fusarium neocosmoporielum*	Proven IFI Localized	Traumatic amputation	NA
48	M	64	BAL (but with multiple site isolates)	NP	Positive	*Aspergillus fumigatus* (sensu stricto)	Proven IFI Localized	Intensive care hospitalization	No response to antifungal treatment
49	M	83	BAL	NP	Positive	*Paecillomyces formosus*	Probable Localized	Imunossupression	NA
50	F	61	Skin	Negative	Positive	*Alternaria infectoria*	Proven Subcutaneous Localized	Solid organ transplant	NA
51	F	26	Skin	Septate hyphae	Positive	*Fusarium solani*/ *Fusarium petroliphum*	Proven IFI Disseminated	Imunossupression (acute myeloid leukemia)	NA
52	M	67	Lung tissue	Compatible with *Paracoccidioides brasiliensis*	NP	*Paracoccidioides brasiliensis*	Proven IFI Localized	Travels to areas of endemic fungi; intensive smoker	NA
53	M	75	Thyroid aspirate	Septate hyphae	Negative	Negative	Proven IFI Localized	NA	NA
54	F	75	BAL	NP	Positive	*Scedosporium boydii*	Probable IFI Localized	Imunossupression	NA
55	M	76	Blood culture	NA	Positive	*Trichosporon ashashii*	Proven IFI Disseminated	Acute myeloid leukemia	NA
56	F	58	Skin	NA	Positive	*Trychophyton rubrum*	Proven Subcutaneous Localized	Solid organ transplant	NA
57	M	63	Brain tissue	NA	Positive	*A. fumigatus* (sensu stricto)	Proven IFI Localized	NA	NA
58	M	56	Skin	Septate hyphae	Positive	*Sporothrix scheckii* (complex)	Proven Subcutaneous Localized	NA	NA
59	M	65	Blood	NP	Positive	*Fusarium dimerum*	Proven IFI Disseminated	Intensive care hospitalization	NA
60	M	61	Cornea scraping tissue	NA	Positive	*Purpureocillium lillacinus*	Probable IFI Localized	NA	NA
61	M	55	BAL	NA	Positive	*Lichteimia racemosa*	Probable IFI Localized	Intensive care hospitalization; mechanic ventilation	Partial response to antifungal treatment
62	F	60	Skin	Septate hyphae	Positive	*Trychophyton rubrum*	Proven Subcutaneous Localized	Solid organ transplant	NA
63	F	66	Bone biopsy/abcess	NP	Positive	*A. fumigatus*(section) *A. felis/parafelis*	Proven IFI Localized	Hemato-oncological patient	NA
64	M	56	Liver tissue	Aseptate hyphae	Negative	Not identified Mucorales	Proven IFI Localized	NA	NA
65	M	58	Skin	Septate hyphae	Positive	*Alternaria infectoria*	Proven Subcutaneous Localized	Solid organ transplant	NA
66	M	63	Skin	Fungal elements	Negative	*Alternaria infectoria*	Proven Subcutaneous Localized	NA	NA
67	M	37	Pleural fluid	NP	Positive	*Aspergillus fumigatus*	Probable IFI Localized	Imunossupression (sarcoidosis)	NA
68	M	67	Skin	Septate hyphae	Positive	*Trychophyton rubrum*	Probable IFI Localized	Solid organ transplant	NA
69	M	64	Nasal sinus tissue	Septate hyphae	Positive	*Aspergillus fumigatus*	Proven IFI Localized	NA	NA
70	M	52	Bone marrow	Intracellular yeasts	Negative	*Histoplasma capsulatum var capsulatum*	Proven IFI Disseminated	HIV/AIDS; travels to areas with endemic fungi	Patient’s death due to fungal infection
71	F	33	Nasal sinus tissue	NP	Positive	*Aspergillus flavus*	Probable IFI Localized	Imunossupression (severe aplastic anemia)	NA
72	F	56	Skin	Septate hyphae	Positive	*Nanniziopsis obscura*	Proven Subcutaneous Localized	Solid organ transplant (liver); diabetes mellitus	No response to antifungal treatment
73	M	56	Tissue (source not referred)	Septate hyphae	Positive	*Aspergillus nidulans*	Proven IFI Localized	HIV/AIDS	NA
74	M	43	Lung tissue	Septate hyphae	Negative	*Cladosporium sphaerospermun*	Proven IFI Localized	NA	NA
75	F	84	BAL/sputum	Septate hyphae	Positive	*Aspergillus niger* (complex)	Probable IFI Localized	Imunossupression; diabetes mellitus	NA
76	F	40	Cervical abscess	Large yeasts	Positive	*Histoplasma capsulatum duboisii*	Proven IFI Localized	HIV/AIDS; travels to areas with endemic fungi	NA

Legend: NP: not performed; NA: information not available; COPD: chronic pulmonary obstructive disease; HIV: human immunodeficiency virus; AIDS: acquired immunodeficiency syndrome; BAL: Bronchoalveolar lavage; IFI: invasive fungal infection; SFI: subcutaneous fungal infection.
